# Genome-wide profiling of human papillomavirus DNA integration in liquid-based cytology specimens from a Gabonese female population using HPV capture technology

**DOI:** 10.1038/s41598-018-37871-2

**Published:** 2019-02-06

**Authors:** Andriniaina Andy Nkili-Meyong, Pamela Moussavou-Boundzanga, Ingrid Labouba, Ismaël Hervé Koumakpayi, Emmanuelle Jeannot, Stéphane Descorps-Declère, Xavier Sastre-Garau, Eric M. Leroy, Ernest Belembaogo, Nicolas Berthet

**Affiliations:** 1 Centre International de Recherches Médicales de Franceville (CIRMF) Department of Zoonosis and Emerging Diseases, Franceville, Gabon; 2 Institut de Cancérologie de Libreville (ICL), Libreville, Gabon; 3 Institut Curie, Department of Biopathology, 26 Rue d’Ulm, 75005 Paris, France; 4 Institut Pasteur, Centre de Bioinformatique, Biostatistique et Biologie Intégrative (C3BI), 25 rue du docteur Roux, 75724 Paris, France; 5 Institut de Cancérologie de Lorraine (ICL), 6 Avenue de Bourgogne, 54519 Vandœuvre-lès-Nancy, France; 6 Institut de Recherches et de Développement (IRD), Maladies Infectieuses et vecteurs: Ecologie, génétique, Evolution et Contrôle (IRD 224 – CNRS 5290 6 UM1- UM2), Montpellier, France; 7 Centre National de Recherche Scientifique (CNRS) UMR3569, 25 rue du docteur Roux, 75724 Paris, France; 8 Institut Pasteur, Unité Environnement et risques infectieux, Cellule d’Intervention Biologique d’Urgence, 25 rue du Docteur Roux, 75724 Paris, France

## Abstract

Human papillomavirus (HPV) is recognised as the cause of precancerous and cancerous cervical lesions. Furthermore, in high-grade lesions, HPV is frequently integrated in the host cell genome and associated with the partial or complete loss of the E1 and E2 genes, which regulate the activity of viral oncoproteins E6 and E7. In this study, using a double-capture system followed by high-throughput sequencing, we determined the HPV integration status present in liquid-based cervical smears in an urban Gabonese population. The main inclusion criteria were based on cytological grade and the detection of the HPV16 genotype using molecular assays. The rate of HPV integration in the host genome varied with cytological grade: 85.7% (6/7), 71.4% (5/7), 66.7% (2/3) 60% (3/5) and 30.8% (4/13) for carcinomas, HSIL, ASCH, LSIL and ASCUS, respectively. For high cytological grades (carcinomas and HSIL), genotypes HPV16 and 18 represented 92.9% of the samples (13/14). The integrated form of HPV16 genotype was mainly found in high-grade lesions in 71.4% of samples regardless of cytological grade. Minority genotypes (HPV33, 51, 58 and 59) were found in LSIL samples, except HPV59, which was identified in one HSIL sample. Among all the HPV genotypes identified after double capture, 10 genotypes (HPV30, 35, 39, 44, 45, 53, 56, 59, 74 and 82) were detected only in episomal form. Our study revealed that the degree of HPV integration varies with cervical cytological grade. The integration event might be a potential clinical prognostic biomarker for the prediction of the progression of neoplastic lesions.

## Introduction

Cervical cancer (CC) is the fourth most common cancer in women worldwide and is the leading cause of cancer deaths among women living in sub-Saharan Africa^1,2^. Among the 200 human papillomavirus (HPV) genotypes identified to date, only 50 genotypes that are capable of infecting the cervical epithelium are classified as “low-risk” (LR) or “high-risk” (HR). LR genotypes are associated with lesions that may regress spontaneously whereas only 16 HPV genotypes classified as HR (16, 18, 31, 33, 34, 35, 39, 45, 51, 52, 56, 58, 59, 66, 68, 70) are explicitly associated with cervical cancer^3,4^.

HPV infection includes integration of the HPV genome into the host genome. This integration leads to the linearization of the HPV genome, usually somewhere the region of the E1 and E2 genes, but can also cause the partial or total deletion of these genes^5,6^. The loss of one or both of these genes leads to the overexpression of the E6 and E7 genes, a condition that contributes to oncogenesis and the progression of low-grade lesions to more severe lesions and ultimately carcinoma. Determining the physical state of the viral genome (integrated or episomal state) and the insertion site may provide a better understanding on how this integration mechanism promotes carcinogenesis^7,8^. HPV integration has been studied using various techniques. The very first approaches included Southern blots and fluorescent *in situ* hybridization^9–11^, but these methods require a large amount of fresh DNA. Various PCR-based approaches requiring less DNA were then developed. Given that the integration of the HPV genome into the host genome generally induces the partial or total deletion of the E1 or E2 genes, the identification of integration status relies on the failure to amplify these genes in their entirety^6,12–14^. More customized molecular techniques such as restriction site PCR (RS-PCR)^15^ and the detection of integrated papillomavirus sequences PCR (DIPS-PCR)^16^ have also been developed, but also cannot distinguish between integrated and episomal forms, or a mixture of these. To overcome this limitation, quantitative real-time PCR that can measure E2/E6 copy numbers have also been developed to determine the ratio of both forms present in a sample^5^. More recently, next-generation sequencing (NGS) has been successfully applied to detecting integration events which consisted in screening for viral-host chimeric junctions. The whole-genome sequencing coupled with read mapping for analysis from DNA extracted from HeLa cell line has been used to determine that the integration event occurred near the *c-myc* oncogene, demonstrating an example of host chromosomal alteration caused by a viral integration associated with an cancer^17^. Similarly, exome sequencing can characterize HPV integration: using the blood from a metastatic cervical carcinoma patient, 1.2 billion reads were generated to detect HPV integration by mapping reads on a reference human genome. This approach identified integrated HPV 18 even in the presence of the episomal form of the same HPV genotype. Another study, using the RNA-seq technique and based on discordant paired-end reads aligned to the viral and the host genomes^18^, revealed an integration rate of 82.3% in cervical squamous cell carcinoma. However, this method can only detect integration sites within coding regions. Finally, the highly sensitive and recent approach combining NGS with capture technology has been used on different types of samples such as snap-frozen^19–21^ or paraffin-embedded^22,23^ tissues from biopsies of carcinoma and adenocarcinoma cases. Still other studies are based on squamous cell carcinoma and cervical adenocarnicoma cell lines^24–26^. The different types of samples and bioinformatics approaches have revealed several integration sites across the human genome with various integration rates increasing with the severity of the lesions^27,28^. Regarding bioinformatics techniques, mapping approaches generally either rely on paired-end reads aligned with the viral and host genomes separately^23,27^ or aligned with an index of combined viral-host genomes^20,29^. Mapping algorithms have also been employed in conjunction with the Smith and Watterman algorithm to increase sensitivity^30^.

In this study, using a double-capture system followed by high-throughput sequencing, we determined the HPV genotype(s) present in liquid-based cervical (LBC) smears performed on an urban Gabonese population. The HPV integration status, as well as the possible integration site(s), were also explored. Our study revealed that the degree of HPV integration varies with the severity of the cytological cervical grade (from atypical to carcinoma cells). Moreover, this integration appeared to be associated with large deletions at the genomic insertion points. The specific integration of HPV samples suggests that HPV integration may be a potential early-stage biological and clinical prognostic biomarker for the prediction of the progression of neoplastic lesions.

## Materials and Methods

### Study population and cervical sample collection

The women participating in this study were recruited during a previous multi-centre cross-sectional study carried out at two hospitals in Libreville, Gabon^31^. The main inclusion criteria were based on cytology and the presence of the HPV16 genotype detected using molecular assays. The characteristics of this recruitment and molecular genotyping of HPV have been described previously^31^. Here, all specimens were divided into five groups according to cytological results: atypical squamous cells of undetermined significance (ASCUS, group 1, n = 13), low-grade squamous intraepithelial lesions (LSIL, group 2, n = 5), atypical squamous cells which do not rule out high-grade squamous intra-epithelial lesions (ASCH, group 3, n = 3), high-grade squamous intraepithelial lesions (HSIL, group 4, n = 7) and carcinoma (group 5, n = 7). This study was approved by the Medical Ethics Committee of Gabon (Consent Number PROT No. 0010/2013/SG/CNE), and was authorised by the Gabonese Ministry of Health (No. 00775/MS/CAB.M/SG/DGS) and the Scientific Committee of the *Centre International de Recherches Médicales de Franceville* (CIRMF). All experiments were performed in accordance with relevant guidelines and regulations and written informed consent was obtained for all included patients.

### Double-capture preparation and sequencing

DNA was quantified using the Quant-iT assay (Invitrogen) and a fixed amount of DNA (500 ng) was fragmented using a Covaris M220 ultrasonicator according to the manufacturer’s instructions. The 450 bp DNA fragments were used to construct a genomic library with the SeqCap EZ Reagent Kit (Roche NimbleGen, Madison, USA) according to the manufacturer’s recommendations. All nucleic acid purification steps were carried out using Agencourt AMpure XP beads (Beckmann Coulter, the Netherlands). After the first steps of end repair, adapter ligation and first pre-capture PCR, a specific HPV capture was performed with SeqCap EZ probes with incubation at 40 °C over the night. After the various stages of washing and amplification of the fragments captured by PCR, a second capture is carried out in order to optimize the enrichment of the HPV fragments and to maximise the fraction of HPV reads in the bioinformatics analyses. A volume of 4 ul of the previous library obtained after the first capture is used as input for the second capture using the same protocol. The final library quality and size were assessed on an Agilent BioAnalyser 2100 (Agilent Technologies, USA) and Illumina Sequencing was conducted using a Miseq benchtop sequencer: 300 cycle runs were performed and 150 nucleotide paired-end reads were obtained.

### Bioinformatics analysis and determination of viral genome integration sites in the human genome

**Figure 1:**
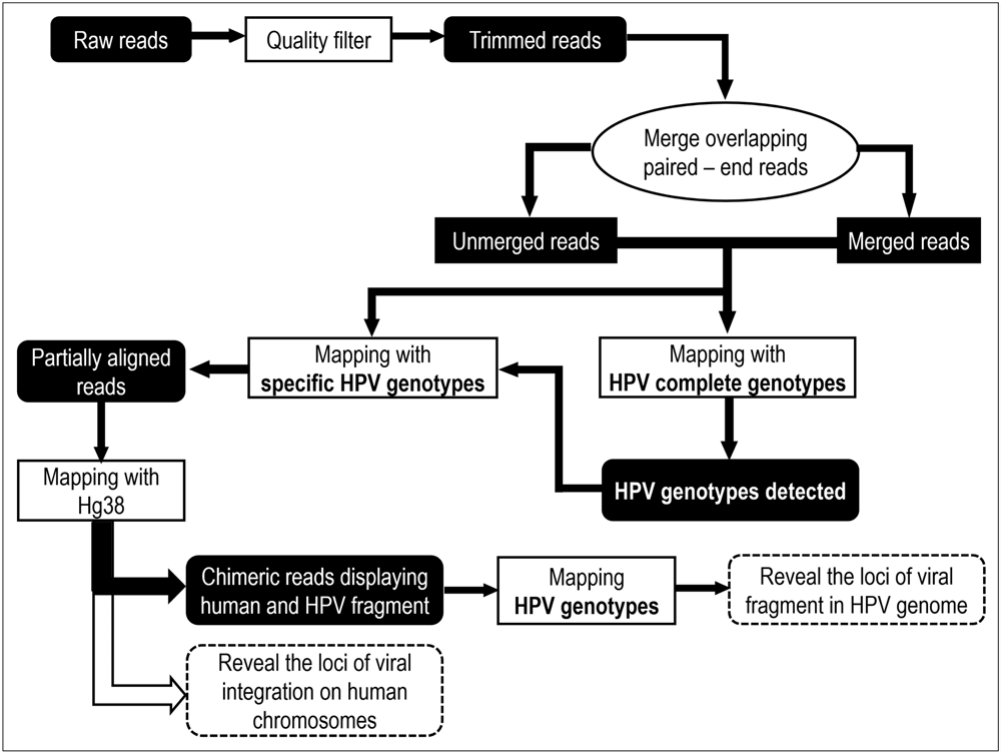
Analysis pipeline for determining integration breakpoint.

**Figure 2:**
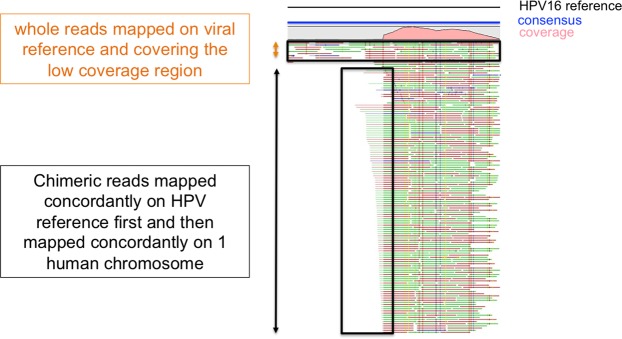
Detection of putative episomal or integrated forms of the virus. An integration event was detected when the coverage of the genomic region between the unaligned part of the read mapped on viral reference was low.

The analysis pipeline is showed in Fig. 1. The quality of the generated reads was initially assessed and filtered using the CLC Workbench 10.0.1 quality control and trimming tool. All filtered paired-end reads were concatenated to obtain longer fragments, and then mapped against complete HPV genomes available in GenBank using CLC Workbench 10.0.1 with at least 40% of the read length aligned and 90% identity (L40/I90). The integration sites were defined as breakpoints with chimeric reads corresponding to human and viral sequences within a concatenated or single read. The process of finding the chimeric reads was based on several successive mappings against viral and human genomes (Fig. 1). A mapping approach instead of BLAST alignment was chosen to save computational time^32^. The chimeric reads have human and viral parts of variable size and, during the successive mappings against viral and human references, potential short sequences may match either reference in a nonspecific manner, leading to false positives. Since no assumptions could be made on the size of the viral and human sequences of chimeric reads, the mapping parameters had to be flexible enough to avoid missing reads with short chimeric fragments, but specific enough to ignore possible false positives due to those tiny chimeric fragments. Therefore, the cut-offs were set to 40% of the entire reads, which had to align with at least 90% identity (L40/I90). Since HPV genotypes are classified in different types based on at least a 10% divergence, the preliminary high-stringency (L90/I90) mapping against a large collection of GenBank’s complete HPV genomes allowed for a rapid distinction between the HPV genotypes involved in potential integration events. The reads were then grouped by HPV genotype. Detection of chimeric reads was then carried out by mapping all reads with the mapping parameters described above: for each identified HPV genotype, the mapped reads were recovered and then mapped against the Hg38 human genome assembly. All groups of mapped reads were individually recovered. For each group, an integration event was determined if (i) all the detected chimeric reads had the same breakpoint position and (ii) the human and viral chimeric fragments had variable lengths, avoiding as far as possible duplicate read issues. Each recovered group was mapped one last time against the sequence of the specific HPV genotype to confirm the presence of chimeric reads and to determine the breakpoint site within the viral genome. A table summarising the chromosomal localisation for each viral integration was generated. Similarly, the previously determined loci of the integration breakpoint were verified by the overlap between the UCSC cytogenetic bands and the chimeric read locations. For samples in which episomal and integrated forms were both observed (Fig. 2), there were two types of mixed profiles, depending on which form predominated based on coverage ratio. A form was determined as predominant if its coverage was at least 10 times higher than the other form’s coverage.

## Results

### Description of the HPV genotypes detected after sequencing the captured fragments

The double-capture system was designed to detect 17 high-risk (HR) HPV genotypes (Table S1). In this study, 35 samples were tested; independently of the cytological grade of the cervical smears tested, 15 HPV genotypes were detected, of which 10 and 5 were HR and low-risk (LR) genotypes, respectively (Tables 1 and S2). Of these 15 HPV genotypes, 6 genotypes (HPV51 + 5 LR) did not belong to the expected detection spectrum of the designed probes and appeared to be captured by homology with the targeted genotypes. The HPV16 genotype was found in 100% (35/35) of samples. However, the integrated status was showed in only 15.4% (2/13), 33.3% (1/3), 57.1% (4/7) and 71.4% (5/7) of ASCUS, ASCH, HSIL and carcinoma samples, respectively. The HPV18 genotype was detected in only 25.7% of samples (9/35) and found integrated in 55.6% of samples (5/9). The HPV33 genotype was found in 65.7% of samples (23/35), but only once in integrated form in an LSIL sample. The other HR-HPV genotypes, most often detected in episomal form, were types HPV56 and HPV59 at 31.4% (11/35) and HPV58 at 37.1% (13/35). However, HPV56 and 58 were found in 81.8% (9/11) and 84.6% (11/13) of neoplasia and carcinoma samples (i.e. LSIL, HSIL, ASCH and carcinomas), whereas HPV59 was mainly found in ASCUS samples (63.6% (7/11)). The most frequent LR-HPV genotype was HPV82 with a rate of 34.2% (12/35 samples), with no particularly predominant cytological grade. The other HR and LR genotypes (HPV30, 39, 44, 45, 53, and 74) were detected in only one or two samples. HPV30, 45 and 74 were found in two samples (HSIL and LSIL) with significantly longer sequence lengths compared with the other samples (Tables 1 and S2).

**Table 1:** Summary of HPV detected for each sample.

Cytological grade	Sample	HPV genotypes
Carcinoma	A9	HPV 16, 33
	B3	HPV 16, 33, 56, 58, 82
	B8	HPV 16, 18, 56, 58
	C6	HPV 16, 18, 58
	C8	HPV 16, 18
	C11	HPV 16, 33
	C12	HPV 16, 18, 39, 58, 82
HSIL	A8	HPV 16, 33, 35, 51, 59
	B1	HPV 16, 33, 56, 58
	B2	HPV 16, 33, 56, 58
	B5	HPV 16, 33, 35, 39, 56, 58, 82
	B12	HPV 16, 30, 33, 39, 45, 56, 58, 74, 82
	C5	HPV 16, 18
	C9	HPV 16, 51
ASCH	A7	HPV 16, 33, 35, 74
	B7	HPV 16, 33, 35, 56, 58, 82
	B9	HPV 16, 18, 33, 56, 58, 82
LSIL	A2	HPV 16, 33, 35, 59
	A11	HPV 16, 33, 35, 45, 51, 59
	B6	HPV 16, 44, 56, 58, 82
	B10	HPV 16, 33, 56, 58, 82
	C7	HPV 16, 51
ASCUS	A1	HPV 16, 33, 35, 59
	A3	HPV 16, 33, 59
	A4	HPV 16, 33, 35, 59, 82
	A5	HPV 16, 33, 35, 59, 82
	A6	HPV 16, 33, 59
	A10	HPV 16, 33, 35, 59
	B4	HPV 16, 33, 56, 58, 82
	B11	HPV 16, 18, 33, 35, 56, 58, 59, 82
	C1	HPV 16, 18
	C2	HPV 16, 53
	C3	HPV 16
	C4	HPV 16
	C10	HPV 16, 18, 33, 51

### Integration of the HPV genome in the human genome

The rate of HPV integration in the host genome varied with cytological grade: 30.8% (4/13), 60% (3/5), 66.7% (2/3), 71.4% (5/7) and 85.7% (6/7) for ASCUS, LSIL, ASCH, HSIL, and carcinomas, respectively (Table 2). The main integrated HPV genotypes, regardless of the cytological grade, were HPV16, 18, 33, 51, 58 and 59. For high cytological grades (carcinomas, HSIL and ASCH), genotypes HPV16 and 18 represented a 92.9% (12/13) of samples. The HPV16 genotype was mainly found in high-grade lesions in 71.4% of samples regardless of the cytological grade. Minority genotypes (HPV33, 51, 58 and 59) were found in LSIL samples, except HPV59 which was identified in one HSIL sample.

**Table 2:** Summary of 21 HPV integration statuses.

Cytological grade	Sample	HPV genotype	Status of integration	Main integration						Supplemental integrations	
				Chromosomal position of integration sites	Size of deleted area (bp)	Chromosomal localisation	Nature of region	Gene or other disrupted sequence	Genes nearby (within 500 kb)	Chromosomal position of integration sites	Chromosomal localisation
Carcinoma	C8	HPV16	Majority	11:4141137.0.4141147	10	11p15.4	non-coding region		RRM1, STIM1		
	B8	HPV18	Majority	13:73970531.0.20:52677001***	N.A.	13q22.1 & 20q13.2	13: intron 20: non-coding region	13: KLF12 20: RP4-715N11.2 (lincRNA)			
	A9	HPV16	Majority	8 :42662221.0.42662224	3	8p11.21	non-coding region				
	B3	HPV16	Minority	3 :167913519	0	3q26.1	non-coding region	LINC01330 (transcribed pseudogene)	GOLIM4, SERPINI1		
	C11	HPV16	Minority	2 :16078561.0.16078617	226	2p24.3	non-coding region	GACAT3 (lincRNA)	MYCN		
	C6	HPV16	Minority	10 :4650152	0	10p15.1	non-coding region				
HSIL	A8	HPV59	Majority	N.D.	N.D.	N.D.					
	B1	HPV16	Majority	11:62609491.0.62678847	69356	11q12.3	intron	GANAB	SCGB1A1, AHNAK, EEF1G, TUT1, RP11-864I4.1, MTA2, ROM1, EML3, B3GAT3, INTS5, GANAB, METTL12, BSCL2, HNRNPUL2, ZBTB3, RP11-831H9.11, LRRN4CL, TTC9C, POLR2G, C11orf98, HNRNPUL2-BSCL2, TAF6L, NXF1, RP11-727F15.14, LBHD, GNG3, TMEM223, STX5, TMEM179B, WDR74, UBXN1, UQCC3, SLC3A2		
	C5	HPV18	Majority	17:62864111.0.62866626	2515	17q24.2	non-coding region		TANC2, MARCH10, MRC2	44398322; 44616177; 45120465	17q21.32; 17q21.33
	C9	HPV16	Minority	14:53825501.0.55712832	1887331	14q22.2 & 14q22.3	non-coding region non-coding region	AL162759.1 (lincRNA)	KTN1, FBXO34, WDHD1, SAMD4A, GCH1, CGRRF1, CDKN3, BMP4* FERMT2, DDHD1, STYX, PSMC6, GNPNAT1, TXNDC16, PTGDR, PELI2, TMEM260**		
	B2	HPV16	Minority	6:126248596	0	6q22.32	non-coding region		CENPW, TRMT11		
	B5	HPV16	Minority	6:107221398.0.107226375	4977	6q21.1	intron	PDSS2	BEND3, C6orf203		
ASCHL	B9	HPV18	Majority	20:5751892	0	20p12.3	intron region	C20orf196	CHGB, TRMT6, MCM8, GPCPD1		
	A7	HPV16	Minority	13:73626724	0	13q22.1	non-coding region	LINC00393 (lincRNA)	KLF12		
SIL	B6	HPV58	Minority	2:1608294	0	2p25.3	non-coding region	AC144450.1 (antisense)	PXDN, MYT1L, SNTG2, TPO		
	C7	HPV51	Minority	2:133183995.0.133185219	1224	2q21.2	intron	NCKAP5		Chr2:3338144; Chr5:5744895	2p25.3; 5p15.32
	B10	HPV33	Minority	10:74594325	0	10q22.1	intron	ADK	KAT6B		
ASCUS	A10	HPV16	Majority	8:24011443.0.24011455	12	8p21.2	non-coding region		STC1		
	B11	HPV18	Majority	21:43348764.0.43348768	4	21q22.3	non-coding region		SIK1, HSF2BP, H2BFS, CRYAA, U2AF1		
	C10	HPV18	Majority	22:41151127.0.41152240	1113	22q13.2	intron	EP300	L3MBTL2, RANGAP1, ZC3H7B, TEF, RBX1, XPNPEP3		
	B4	HPV16	Minority	5:147283983.0.147285227	1244	5q33.1	intron	STK32A	DPYSL3, PPP2R2B		

N.D. Not determined; *deleted genes between breakpoint of HPV genome; **genes nearby; lncRNA: long non-coding RNA; ***chromosomal translocation.

Among all the HPV genotypes identified after double capture, 10 genotypes (HPV30, 35, 39, 44, 45, 53, 56, 59, 74 and 82) were detected only in episomal form; no virus-human chimeric sequences were detected after mapping them on reference sequences. For samples in which episomal and integrated forms were both observed, there were two types of mixed profiles, depending on which form predominated based on coverage ratio. One mixed profile Epi/Int showed a predominance of the episomal form compared with the integrated form, whereas for the other mixed profile Int/Epi, the integrated form predominated the episomal form. The mixed Int/Epi HPV profile was found in 47.6% (10/21) of samples, whereas the mixed Epi/Int profile was observed in 52.8% (11/21) of samples. In high-grade lesions (carcinomas, HSIL and ASCH), both profiles were found in equivalent proportions, whereas in all LSIL samples, only the Epi/Int profile was observed. HPV16 and 18 were respectively found in 40% and 50% of Int/Epi samples. HPV16 was involved in 75% of Epi/Int samples, whereas HPV18 was not detected in any Epi/Int samples.

### Chromosomal localisation of viral genome integration

For all cytological grades, viral integration in the host genome was sometimes followed by deletion of either part of the viral genome or part of the host genome at the integration site. The breakpoint sites in the viral genome, before integration, were located in one of four viral genes (E1, E2, L1, and L2) independently of the cytological grade. A breakpoint site in the E2 gene represented about 40% (12/30) of integrations followed by E1, L1 and L2 with 26.7% (8/30), 23.3% (7/30) and 10% (3/30), respectively. The partial or total deletion of the E2 gene was found in 84.2% of cases (16/19), with the first breakpoint site being located upstream or downstream from the gene and the second site being located either within or further downstream from the gene. The E2 deletion was found in all carcinomas, ASCH and LSIL samples and in 75% and 80% of ASCUS and HSIL samples, respectively. The breakpoint sites were found outside the E2 gene in only two samples; namely either within the E1 gene or between the L1 and L2 genes for ASCUS and HSIL samples.

Deletion of host genome sequences was observed at the integration sites in 57.9% of samples (11/19) (Table 2). The size of the deletion varied from 3 to 1.88 × 10^6^ nucleotides for carcinoma (A9) and HSIL (C9) samples. In the other cases (8/19), the viral genome integrated without any modifications to the host genome (Table 2). As shown in previous studies^33^, we did not detect any preferential integration sites, whether in a chromosome or at a particular locus. Of the 21 samples, 28.6% (6/21) of the integration sites were found in a unique chromosome, whereas the other integration sites were found at least twice in a single chromosome but in different loci (Table 2). Chromosome 2 showed the most integrations, with three integrations located at different loci, in two low-grade lesions (LSIL - 2p25.3 and 2q21.2) as well as in a carcinoma (2p24.3) (Table 2). Only one case of two integration sites was found in a HSIL sample, and the sites were located in two different loci (C9 - 14q22.2 and 14q22.3), with the deletion of 1.8 × 10^6^ nucleotides between these two sites. Moreover, as shown in previous studies^34,35^, only one case of chromosomal translocation between chromosomes 13 and 20 (B8 - 13q22.1 & 20q13.2) of an HPV18 integration was observed in a carcinoma. Finally, we observed multiple integration events with the same genotype in only two samples, LSIL and HSIL: either on the same chromosome but in different loci (C5 - 17q24.2 *versus* 17q21.32 to 17q21.33), or in a different locus on the same chromosome or on a different chromosome (C7 - 2q21.2 *versus* 2p25.3 and 5p15.32).

The detailed analysis of the localisation of HPV integration in each locus previously identified showed that in 36.4% of samples (8/22), HPV integration occurred in an intron whereas in 40.8% of samples (9/22) it occurred in a non-coding region. In parallel, in 22.8% of cases (5/22), the integration occurred either in a long non-coding RNA (3/22) or in an antisense RNA or a putative transcript of a pseudo-gene (2/22). Except for two carcinoma samples (B8 and C7), there was always a group of genes found in close proximity to the HPV integration site (+/−500 kb), among which some may be involved in cell division or are oncogenes such as MYCN (C11) or RRM1 (C8)^36,37^. Similarly, HPV integration appeared to have occurred in the intron of a gene involved in cellular processes such as the Kruppel-like factor 12 (KLF12) or the coding gene for the E1A binding protein p300 in a carcinoma (B8) or an ASCUS (C10), respectively. Finally, in this study, only two HSIL samples were associated with a large loss of a chromosomal region (60 and 1800 kb respectively for cases B1 and C9). In each sample, the loss was accompanied by the deletion of several entire genes (introns and exons) or of eight exons for the UBXN1 gene for example (B1) (Table 2).

## Discussion

Here, we used a double-capture system to investigate HPV integration sites in the human genome on cervical smears with precancerous and cancerous lesions (LSIL and HSIL) or undetermined atypical cells (ASCUS and ASCH). Previous studies based on cervical biopsies have demonstrated viral genome integration in the early stages of lesion development^21,27^. For example, a study of high-throughput viral integration detection (HIVID) identified 3667 integration breakpoints of HPV with integration rates comprised between 44.4% and 71.4% according to the clinicopathological stage considered (CIN1 to CIN3)^21^. Furthermore, these integration rates increased significantly with the severity of neoplastic lesions. However, 87% of these integration points (reported by Hu *et al*.) may, in fact, be experimental or computational artefacts^38^. The various studies on integration sites based on a double-capture system followed by high-throughput sequencing of the captured fragments also report variable integration rates in precancerous lesions as well as invasive carcinomas^23,27^. Nevertheless, although integration rates may vary among studies, they are significantly higher in CIN3 than in CIN1 and CIN2. It is difficult to compare these data with those obtained in our study, because we did not use cervical biopsies, but LBC smears on which only cytological analyses were performed. However, although it is not possible to attain perfect correspondence between histological and cytological data, HSIL and ASCH cases are usually indicative of high-grade lesions and are comparable to the CIN3 stage, and LSIL cases to the CIN1 stage. Based on our cytological data, we observed the same pattern of increased integration with increased lesion severity (60%, 85.7% and 85.7% respectively for LSIL, HSIL and carcinomas). Unlike previous studies, we also analysed smears with undetermined atypical cells (ASCUS). Despite the limited number of these samples, the integration rate found in ASCUS was even lower than in the LSIL samples, with respectively 30.7% and 60%. Altogether, our results tend to confirm that the viral genome integration process is an event that occurs very early in the development of neoplastic lesions^39^. However, of four ASCUS patients in whom integration was revealed, two of them died less than four years after the first cervical cancer investigations and one developed precancerous lesions without dying. The last patient has not been located, but it is very probable that she did not develop cervical cancer because she does not appear in the pathology monitoring program that is currently being conducted in Gabon^40^. The other located patients with ASCUS status in which no integration was found have not died or developed high-grade precancerous lesions. As described in literature, these data appear to suggest that some cytological analyses did not reveal high-grade (HSIL) precancerous lesions in patients after performing the tests^41^. Although the number of LSIL cases in this study was small compared with the other cytological grades, the integrated HPV genotypes were neither HPV16 nor 18, two genotypes that were involved in many high-grade lesion cases in our cohort. In addition, the genotypes identified in our LSIL (HPV33, 51, and 58) cases have been associated with carcinomas in other studies, albeit not the most frequently found genotypes. All these data suggest that the integration of the HPV genome is not a rare event and that it already occurs at a low-grade lesion stage. However, given that HPV33, 51 and 58 genotypes were never found integrated in more severe grades, it is possible that the size of our high-grade and carcinoma cohort is too small to reveal them. Other studies led in Gabon have confirmed that genotypes HPV16 and 18 are the two main genotypes found in carcinomas, followed by genotypes HPV33 and 58, but in much lower proportions^42,43^. These studies also demonstrate that the minor genotypes such as HPV33 and 58 can also be found in co-detection with HPV16 or 18, and therefore may not necessarily be responsible for the cancerous lesion Indeed, given that cervical smears can collect a large number of cells from the cervix, and not only from the area of the cancerous lesion, it is not surprising to find multi-infections of HPV^42,43^, unlike a biopsy which mainly collect cells infected by a single genotype from the lesion. In this study, there was no evidence that these minor genotypes associated with HPV16 and/or 18, particularly in high-grade lesions, were integrated into human genome. In any case, whatever the cytological grade considered, no simultaneous integration of different genotypes has been found.

**Figure 3:**
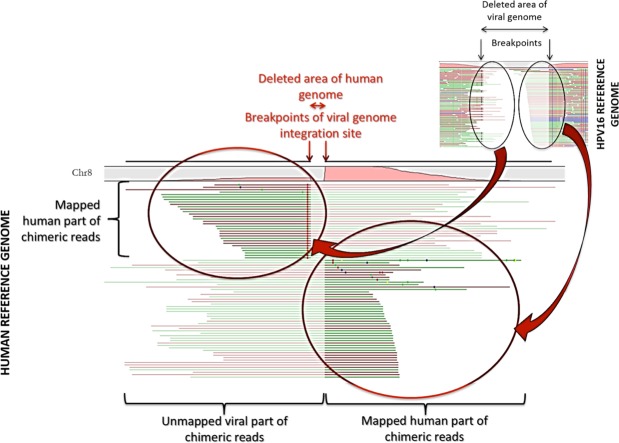
Chimeric reads from mapping against viral reference are next mapped again human reference. The reads that aligned are in solid colour and the unaligned part is faded. If those chimeric reads mapped concordantly on the same human chromosome, then an integration event was called for this chromosome. Therefore the detection of integration events is based on chineric reads, mapped concordantly on viral reference, associated with low coverage and mapping on a single human chromosome.

In this study, the detection of integration sites was based on successive read mappings against viral references and then host genome (Fig. 3). This method successfully identified the integration position within the host genome and which part of the viral genome was involved. However, issues on the specificity and sensitivity of this method must be addressed. Other studies on HPV integration in the human genome are also based either on mapping tools or sequence alignment tools such as BLASTN or BLAT^29,44^. Alignment-based methods seem *prima facie* unbiased regarding chimera composition because no assumptions, concerning the size of the host and viral sequences in chimeric reads, are made. Therefore, the risk of missing host/viral chimeric reads resulting from integration sites should be low, regardless of the size of human and viral sequences of these chimera. Nevertheless, querying a database comprising only one organism introduces a bias. For instance, BLASTN is designed to detect similarities and is likely to find distant homology between unrelated sub-sequences, thereby ‘forcing’ the alignment to some extent. The same “artificial” human reads generated from Hg38 (Table 3) were also used for an alignment using BLAST (evalue = 0.0001) and HPV references from Table S1. This alignment resulted in 136,245/58,333,246 (2.3%) false positives. On the other hand, mapping methods may be questionable when it comes to specificity and working with genome sizes with different orders of magnitude. In fact, regarding the mapping parameters, especially the identity percentage and the minimum length of a read required to consider the latter as mapped, the reads may be assigned to the wrong genome^23^. Looking for chimeric reads by mapping in local alignment may inadvertently lead to finding a short sub-sequence that maps to both organisms. Our analysis showed that the decrease in the minimum length in either local or global mode for mapping parameters (CLC genomics), expressed in percentage or with a fixed number of bases, led to more viral reads being mapped on the Hg 38 genome (Table 3), increasing the rate of false positives. Consequently, although rare, cases mistaking a viral sub-sequence for a human one (or vice-versa) may lead to confusion regarding integration events. Therefore, the very stringent local mapping parameters used in this study were likely to limit the risk of false positives (Table 3). By choosing carefully the parameters, our mapping approach, more than saving computational time, allowed for keeping low the potential rate of false positives that can be returned by alignment methods. However, these stringent parameters can either be the reason for a lower integration rate than in other studies or may mask other events, such as multiple integration, by eliminating chimeric reads that are weakly represented. For instance, we observed only two cases of multiple integrations with the same genotype on the same chromosome. Lastly, as described in the literature^21,23^, micro-homologies were systematically observed in regions adjacent to integration sites of the viral genome in the host’s genome whatever the mapping parameters used (data not shown).

**Table 3:** Mapping parameters used for finding chimeric reads with CLC genomics workbench 10. The reads used for these comparisons were produced using a sequencing read generator tool (ART_ILLUMINA v2.5.8).

Tool parameters	CLC mapping		human reads-HPV references	HPV reads-human reference
•match score = 1	Gobal alignment	90% identity	4,572,369 (7.8%)	1,448,604 (99.9%)
•mismatch cost = 2		**40% length**		
•Linear gap cost of insertion/deletion		90% identity	4,576,999 (7.8%)	1,448,607 (99.9%)
•Insertion cost = 3		**30% length**		
•Deletion cost = 3	Local alignment	90% identity	10,226 (0.01%)	6,365 (0.4%)
		**40% length**		
		90% identity	49,806 (0.08%)	17,688 (1.2%)
		**30% length**		
	Total reads	58,333,246 (human)	1,448,670 (HPV)	

Finally, owing to the greater sensitivity in the detection of integration phenomena, the analysis of our data regarding integration shows that they can be classified into two types of profiles: one in which the integrated form predominates a residual episomal form, and those for which the episomal form dominates the integrated form. This residual episomal- predominant form (Epi/Int) can be due to the presence of several cells infected by the same HPV genotype, but at a different stage. Additionally, the Epi/Int form could be the result of smaller number of cells which harbours a higher number of copies compared with the cells with an integrated form where there would be only one copy per cell. On the other hand, when the integrated form of the genotype is predominant (Int/Epi), the cell is probably already in the clonal multiplication phase due to the disruption in cellular functions linked to the expression of genes E6 and E7. The development of cervical cancer is a process that requires several stages in which many genetic alterations intervene, activating many cellular oncogenes or inactivating tumour suppressor genes. In either case, the expression of the HPV viral oncogenes E6 and E7 is required during all stages of tumour progression. The integration of the viral genome often leads to the partial deletion of viral genes such as E1, E2, L1, and L2. In our study, all observed integrations were accompanied by the loss of viral sequences, with deletion of the E2 ORF in 84.2% of the cases. As suggested in other studies, the loss of this region may induce the deregulation of the expression of oncogenes E7 and E6^45^. However, the analysis of these integration sites in the human genome cannot explain the link between viral integration and the cellular modifications that we observed, except in some samples in which integration occurred close to genes known to be involved either in the cellular division process (RRM1) or to act as proto-oncogenes (c-myc)^33^. Transcriptomic analyses to confirm these modifications in expression would be difficult to perform because our initial samples are a mix of healthy and transformed cells obtained from a cervical smear.

To conclude, the integration rate of HPV genomes in the host genome varies according to the cytological grade considered, as observed on biopsies in other studies. The possibility of detecting these integration events from smears performed in liquid-based environments has many advantages for developing countries or for those whose at-risk populations have little or limited access to a health system. Smears are easy to perform and do not require any particular conditions during their transport to the analysis laboratory. In addition to other molecular investigations, the early screening for HPV integration in low-grade or atypical lesions can be used as a clinical prognostic biomarker for better prediction of the progression of these neoplastic lesions.

## Supplementary information

Supplementary Dataset 1

## Data Availability

All raw data produced during this study are available Under Accession Number: SUB4880803.
