# Supplementary material for: Genome-wide profiling of human papillomavirus DNA integration in liquid-based cytology specimens from a Gabonese female population using HPV capture technology
Source: Sci Rep. 2019 Feb 6;9:1504. doi: 10.1038/s41598-018-37871-2 (PMC6365579; doi:10.1038/s41598-018-37871-2)
Supplement: Supplementary file 1 — Supplementary Dataset 1 [file 41598_2018_37871_MOESM1_ESM.docx]

**Genome-wide profiling of human papillomavirus DNA integration in liquid-based cytology specimens from a Gabonese female population using HPV capture technology**

Andriniaina Andy Nkili-Meyong^1*^, Pamela Moussavou-Boundzanga^1^*, Ingrid Labouba^1^, Ismaël Hervé Koumakpayi^2^, Emmanuelle Jeannot^3^, Stéphane Descorps-Declère^4^, Xavier Sastre-Garau^5^, Eric M. Leroy^1,6^, Ernest Belembaogo^2^, Nicolas Berthet §^1,7,8^

**Supplemental tables**

**Table S1 | List of HPV reference sequences used for the design of capture probes.**

| **HPV genotype**  **(sub-lineage)** | **Size (bp)** | **Accession number** |
| --- | --- | --- |
| HPV 16 (A1) | 7904 | K02718 |
| HPV 16 (A2) | 7904 | AF536279 |
| HPV 16 (B1) | 7906 | AF536180 |
| HPV 16 (C) | 7904 | AF472509 |
| HPV 18 | 7857 | AY262282 |
| HPV 6 | 7902 | X00203 |
| HPV 11 | 7931 | M14119 |
| HPV 31 | 7912 | J04353 |
| HPV 33 | 7909 | M12732 |
| HPV 35 | 7879 | X74477 |
| HPV 39 | 7833 | M62849 |
| HPV 45 | 7858 | X74449 |
| HPV 52 | 7942 | X74481 |
| HPV 56 | 7844 | X74483 |
| HPV 58 | 7824 | D90400 |
| HPV 59 | 7896 | X77858 |
| HPV 66 | 7824 | U31794 |
| HPV 68 | 7822 | KC470269 |
| HPV 69 | 7700 | AB027020 |
| HPV 82 | 7871 | AB027021 |

**Table S2 | Summary of HPV detected for each sample with the consensus sequence length obtained per genotype. High-risk genotypes shown in bold.**

|  |  |  | **HPV genotypes** | | | | | | | | | | | | | | |
| --- | --- | --- | --- | --- | --- | --- | --- | --- | --- | --- | --- | --- | --- | --- | --- | --- | --- |
|  |  | **Sample number** | **HPV 16** | **HPV 18** | HPV 30 | **HPV 33** | **HPV 35** | **HPV 39** | HPV 44 | **HPV 45** | **HPV 51** | HPV 53 | **HPV 56** | **HPV 58** | **HPV 59** | HPV 74 | HPV 82 |
| **Cytological grade** | **Carcinoma** | **A9** | 7907 |  |  | 7912 |  |  |  |  |  |  |  |  |  |  |  |
|  |  | **B3** | 7907 |  |  | 2240 |  |  |  |  |  |  | 7740 | 7773 |  |  | 6222 |
|  |  | **B8** | 7904 | 4590 |  |  |  |  |  |  |  |  | 6307 | 5764 |  |  |  |
|  |  | **C6** | 7906 | 2918 |  |  |  |  |  |  |  |  |  | 7817 |  |  |  |
|  |  | **C8** | 7524 | 6140 |  |  |  |  |  |  |  |  |  |  |  |  |  |
|  |  | **C11** | 6051 |  |  | 7911 |  |  |  |  |  |  |  |  |  |  |  |
|  |  | **C12** | 7904 | 6209 |  |  |  | 7844 |  |  |  |  |  | 7820 |  |  | 7862 |
|  | **HSIL** | **A8** | 7779 |  |  | 7913 | 4424 |  |  |  | 7172 |  |  |  | 7498 |  |  |
|  |  | **B1** | 7904 |  |  | 2647 |  |  |  |  |  |  | 7817 | 7591 |  |  |  |
|  |  | **B2** | 7904 |  |  | 3800 |  |  |  |  |  |  | 7792 | 7729 |  |  |  |
|  |  | **B5** | 7904 |  |  | 7911 | 7855 | 6911 |  |  |  |  | 7844 | 7824 |  |  | 7860 |
|  |  | **B12** | 7904 |  | 4831 | 7911 |  | 7833 |  | 5613 |  |  | 7841 | 7820 |  | 2788 | 7862 |
|  |  | **C5** | 7904 | 7823 |  |  |  |  |  |  |  |  |  |  |  |  |  |
|  |  | **C9** | 7908 |  |  |  |  |  |  |  | 4013 |  |  |  |  |  |  |
|  | **ASCH** | **A7** | 7910 |  |  | 7912 | 5204 |  |  |  |  |  |  |  | 7687 |  |  |
|  |  | **B7** | 7909 |  |  | 2143 | 7879 |  |  |  |  |  | 7744 | 7780 |  |  | 5546 |
|  |  | **B9** | 7904 | 7808 |  | 7332 |  |  |  |  |  |  | 5438 | 6523 |  |  | 3664 |
|  | **LSIL** | **A2** | 5098 |  |  | 7912 | 1773 |  |  |  |  |  |  |  | 5274 |  |  |
|  |  | **A11** | 7906 |  |  | 7913 | 7879 |  |  | 7454 | 2700 |  |  |  | 7898 |  |  |
|  |  | **B6** | 7904 |  |  |  |  |  | 2389 |  |  |  | 7835 | 7817 |  |  | 7820 |
|  |  | **B10** | 7904 |  |  | 7912 |  |  |  |  |  |  | 7844 | 7824 |  |  | 7695 |
|  |  | **C7** | 7906 |  |  |  |  |  |  |  | 7519 |  |  |  |  |  |  |
|  | **ASCUS** | **A1** | 4479 |  |  | 7913 | 1920 |  |  |  |  |  |  |  | 4602 |  |  |
|  |  | **A3** | 1296 |  |  | 7915 |  |  |  |  |  |  |  |  | 1396 |  |  |
|  |  | **A4** | 5151 |  |  | 7913 | 2357 |  |  |  |  |  |  |  | 6094 |  | 7193 |
|  |  | **A5** | 4429 |  |  | 7913 | 1681 |  |  |  |  |  |  |  | 5091 |  | 1062 |
|  |  | **A6** | 2754 |  |  | 7913 |  |  |  |  |  |  |  |  | 2154 |  |  |
|  |  | **A10** | 7809 |  |  | 7913 | 4517 |  |  |  |  |  |  |  | 7226 |  |  |
|  |  | **B4** | 7905 |  |  | 3943 |  |  |  |  |  |  | 6903 | 7473 |  |  | 3240 |
|  |  | **B11** | 7046 | 6830 |  | 7913 | 7010 |  |  |  |  |  | 7663 | 7817 | 7843 |  | 6215 |
|  |  | **C1** | 5197 | 1449 |  |  |  |  |  |  |  |  |  |  |  |  |  |
|  |  | **C2** | 6995 |  |  |  |  |  |  |  |  | 4499 |  |  |  |  |  |
|  |  | **C3** | 7820 |  |  |  |  |  |  |  |  |  |  |  |  |  |  |
|  |  | **C4** | 5858 |  |  |  |  |  |  |  |  |  |  |  |  |  |  |
|  |  | **C10** | 7770 | 7857 |  | 7913 |  |  |  |  | 3731 |  |  |  |  |  |  |
